# Understanding heart failure with preserved ejection fraction: where are we today?

**DOI:** 10.1007/s12471-016-0810-1

**Published:** 2016-02-24

**Authors:** L. van Heerebeek, W. J. Paulus

**Affiliations:** Institute for Cardiovascular Research VU (ICaR-VU), VU University Medical Center Amsterdam, Amsterdam, The Netherlands; Department of Cardiology, Onze Lieve Vrouwe Gasthuis, Amsterdam, The Netherlands

**Keywords:** Heart failure, Diastole, Inflammation, Endothelial dysfunction

## Abstract

Heart failure with preserved ejection fraction (HFpEF) represents a complex and heterogeneous clinical syndrome, which is increasingly prevalent and associated with poor outcome. In contrast to heart failure with reduced ejection fraction (HFrEF), modern heart failure pharmacotherapy did not improve outcome in HFpEF, which was attributed to incomplete understanding of HFpEF pathophysiology, patient heterogeneity and lack of insight into primary pathophysiological processes. HFpEF patients are frequently elderly females and patients demonstrate a high prevalence of non-cardiac comorbidities, which independently adversely affect myocardial structural and functional remodelling. Furthermore, although diastolic left ventricular dysfunction represents the dominant abnormality in HFpEF, numerous ancillary mechanisms are frequently present, which also negatively impact on cardiovascular reserve. Over the past decade, clinical and translational research has improved insight into HFpEF pathophysiology and the importance of comorbidities and patient heterogeneity. Recently, a new paradigm for HFpEF was proposed, which states that comorbidities drive myocardial dysfunction and remodelling in HFpEF through coronary microvascular inflammation. Regarding the conceptual framework of HFpEF treatment, emphasis may need to shift from a ‘one fits all’ strategy to an individualised approach based on phenotypic patient characterisation and diagnostic and pathophysiological stratification of myocardial disease processes. This review will describe these novel insights from a pathophysiological standpoint.

## Introduction

Heart failure with preserved ejection fraction (HFpEF) currently accounts for more than 50 % of all heart failure patients, and its prevalence relative to heart failure with reduced ejection fraction (HFrEF) is rising at a rate of approximately 1 % per year, while HFpEF patients have only slightly lower mortality rates than HFrEF patients [1]. By 2020, the prevalence of HFpEF will exceed 8 % of persons older than 65 years of age and the relative prevalences of HFpEF and HFrEF are predicted to be 69 and 31 %, turning HFpEF into the most common heart failure phenotype [1]. HFpEF is diagnosed in the presence of heart failure signs and/or symptoms, preserved systolic left ventricular (LV) function, with an LV ejection fraction (LVEF) > 50 % and LV end-diastolic volume index < 97 ml/m^2^ with evidence of diastolic LV dysfunction [2]. In contrast to HFrEF, modern heart failure pharmacotherapy did not improve the prognosis in HFpEF and all large randomised HFpEF trials [3–7] have yielded neutral results. These neutral results of recent HFpEF trials were attributed to an incomplete understanding of HFpEF pathophysiology, suboptimal study designs, inadequate diagnostic criteria or statistical power, patient heterogeneity and poor matching of therapeutic mechanisms and primary pathophysiological processes [8]. In the past decade, clinical and translational research provided exiting novel insights into HFpEF pathophysiology and the importance of comorbidities and patient heterogeneity, which could be of great interest for the design and interpretation of future trials as will be discussed in this review.

## Pathophysiology of diastolic LV dysfunction in HFpEF

**Fig. 1:**
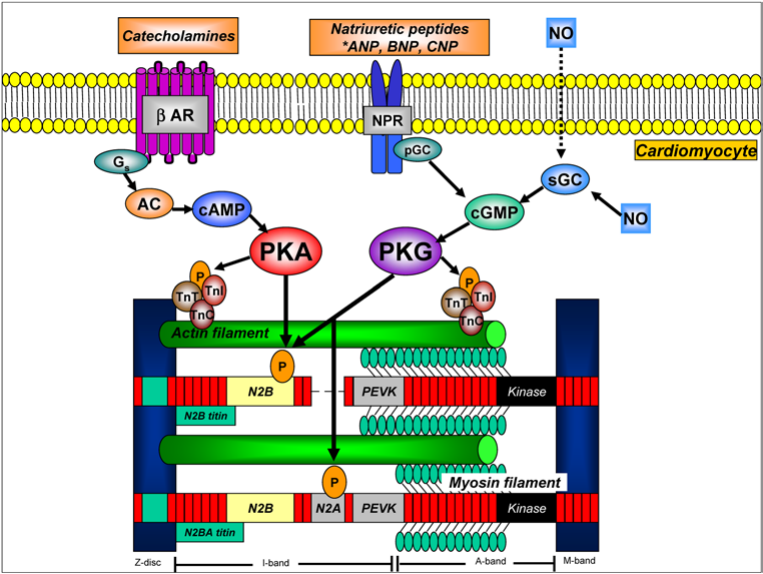
Cardiomyocyte cAMP and cGMP signalling pathways involved in myofilament regulation and titin-based stiffness. Stimulation of β-ARs activates Gs -AC-mediated generation of cAMP, which stimulates PKA activity. cGMP is generated from activation of sGC by NO and from activation of pGC by NPs. cGMP stimulates PKG activity. Both PKA and PKG induce lusitropic effects through phosphorylation of TnI, and lower cardiomyocyte stiffness through phosphorylation of the titin N2B segment. Circled P’s indicate phosphorylatable sites. *AC* adenylyl cyclase, *βAR* beta-adrenergic receptor, *cAMP* cyclic adenosine monophosphate, *G* G-stimulatory protein, *NPR* natriuretic peptide receptor, *PEVK* unique sequence rich in proline, glutamic acid, valine and lysine

**Fig. 2:**
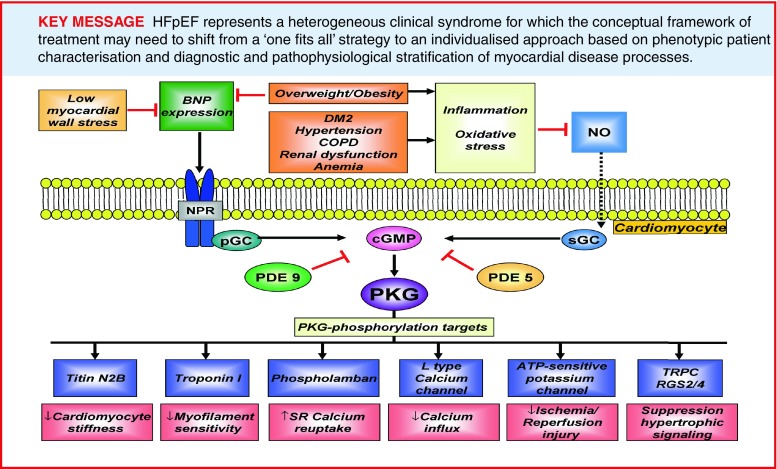
Mechanisms explaining downregulation of myocardial cGMP-PKG signalling in HFpEF. *PDE5* phosphodiesterase type 5, *PDE9* phosphodiesterase type 9, *SR* sarcoplasmic reticulum, *RGS2/4* regulator of G-protein signalling 2 and 4

Under physiological conditions, LV pressure rapidly decreases after systole, allowing fast diastolic LV filling at maintained low filling pressures. Diastolic LV dysfunction in HFpEF is evident from slow LV relaxation and elevated diastolic LV stiffness, which increase diastolic filling pressures and limit cardiac performance at rest, during atrial pacing and exercise [9, 10]. Insight into the pathophysiology of diastolic LV dysfunction in HFpEF has long been missing because of a lack of myocardial tissue obtained from patients with HFpEF. Over the past decade, several groups of investigators were able to obtain myocardial tissue from HFpEF patients revealing specific alterations in myocardial structure, function and intramyocardial signalling, which were relevant to the concentric LV remodelling and diastolic LV dysfunction characteristically observed in patients with HFpEF (Table 1; [11–18]). Structural alterations consisted of cardiomyocyte hypertrophy [12, 13] and varying degrees of myocardial interstitial fibrosis [11–13, 17, 18] and capillary rarefaction [18], whereas functional alterations included increased cardiomycyte stiffness [11–15]. The same studies also demonstrated abnormal intramyocardial signalling, which was evident from endothelial cells expressing adhesion molecules [13, 16], inflammatory cells secreting profibrotic transforming growth factor β (TGF-β) [16] oxidative stress increasing nitrotyrosine content [15, 16] and downregulation of myocardial cyclic guanosine monophosphate (cGMP)-protein kinase G (PKG) signalling [15]. Myocardial cGMP-PKG signalling is crucial for normal cardiovascular physiology, inhibiting maladaptive hypertrophy and enhancing cardiomyocyte compliance through PKG-mediated phosphorylation of the sarcomeric protein titin [19, 20]. Cardiomyocyte stiffness is mainly determined by the elastic sarcomeric protein titin, which functions as a bidirectional spring, responsible for early diastolic recoil and late diastolic distensibility [19]. Titin-based cardiomyocyte stiffness results from dynamic changes in expression of stiff (N2B) and compliant (N2BA) isoforms, from isoform phosphorylation status and from oxidative changes of the N2B segment [19]. Phosphorylation of titin by protein kinase A (PKA) and PKG increase its compliance, thereby acutely lowering cardiomyocyte stiffness (Fig. 1; [11–15]). Various studies, which procured endomyocardial tissue from patients with HFpEF, HFrEF and aortic stenosis, demonstrated significantly stiffer cardiomyocytes in HFpEF than in HFrEF and aortic stenosis patients [11–15]. This increased cardiomyocyte stiffness was related to increased titin N2B isoform expression, relative to HFrEF [12], and to reduced phosphorylation of titin [14]. Hypophosphorylation of titin resulted from lower myocardial PKG activity and reduced myocardial cGMP concentration in HFpEF compared with HFrEF and aortic stenosis [15]. The generation of the second messenger molecule cGMP results from activation of soluble guanylate cyclase (sGC) by nitric oxide (NO) and from activation of particulate GC (pGC) by natriuretic peptides (NPs) (Fig. 2; [20]). Once generated, cGMP activates PKG allowing PKG-mediated phosphorylation of a vast number of target proteins, exerting a wide range of downstream effects such as enhanced reuptake of calcium (Ca2+) into the sarcoplasmic reticulum, inhibition of Ca2+ influx, suppression of hypertrophic signalling through inhibition of G-protein coupled receptors and the transient receptor potential canonical channel (TRPC), inhibition of ischaemia-reperfusion injury through phosphorylation of the ATP-sensitive potassium channel and stimulation of LV relaxation and LV distensibility by phosphorylation of troponin I (TnI) and the titin N2B segment (Fig. 2; [19, 20]). Downregulation of myocardial cGMP-PKG signalling in HFpEF is related to reduced myocardial brain-type NP (BNP) expression and increased microvascular inflammation and oxidative stress, which impair both the NP-cGMP and NO-cGMP axes (Fig. 2; [15]). Reduced myocardial BNP expression in HFpEF could have resulted from a number of factors, including concomitant obesity and insulin resistance, which lower myocardial BNP expression [21] and concentric LV remodelling/hypertrophy, which reduces both systolic and diastolic LV wall stress [22]. In addition, low myocardial BNP expression in HFpEF could also have resulted from increased expression of phosphodiesterase (PDE) type 9, which breaks down cGMP specifically generated through the NP-pGC axis [23]. Impaired NO-cGMP signalling could have resulted from the increased inflammation and oxidative stress observed in HFpEF, which was inferred from the high prevalence of comorbidities such as hypertension, obesity and diabetes mellitus type 2 (Fig. 2; [15]).

**Table 1:** Specific alterations in myocardial structure, function and intramyocardial signalling demonstrated in HFpEF patients

Structural alterations	Functional alterations	Intramyocardial signalling alterations
Cardiomyocyte hypertrophy	Increased cardiomyocyte stiffness	Endothelial cells expressing adhesion molecules
Interstitial fibrosis	Impaired cardiomyocyte relaxation	Inflammatory cells secreting TGF-β
Capillary rarefaction		Oxidative stress increasing nitrotyrosine content
		Downregulation of myocardial cGMP-PKG signalling

## Comorbidities in HFpEF

**Fig. 3:**
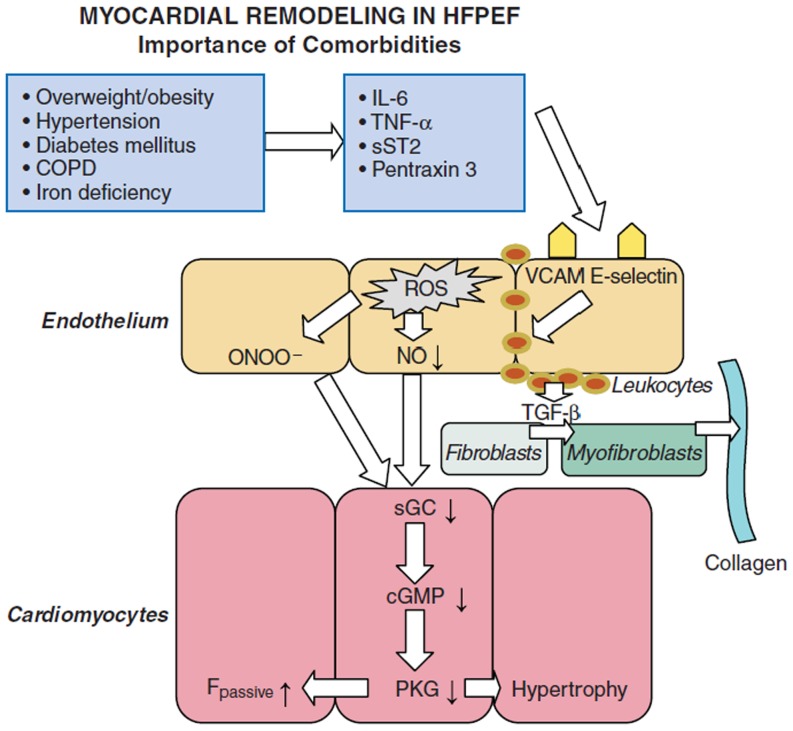
Comorbidities drive myocardial dysfunction and remodelling in HFPEF. *IL-6* interleukin-6; *sST2* soluble ST2; *TNF-α* tumour necrosis factor alfa; *VCAM* vascular cell adhesion molecule. Modified with permission from [28]

HFpEF patients are generally older, more often female and have a high prevalence of cardiovascular and non-cardiovascular comorbidities, such as obesity, metabolic syndrome, diabetes mellitus type 2, salt-sensitive hypertension, atrial fibrillation (AF), chronic obstructive pulmonary disease, anaemia and renal dysfunction [24–26]. Systemic inflammation and endothelial dysfunction are important hallmarks of these comorbidities and are also importantly involved in HFpEF pathophysiology [16, 27]. Myocardial inflammation was shown to contribute to extracellular matrix changes in HFpEF and both myocardial collagen and the amount of inflammatory cells correlated with diastolic LV dysfunction [16]. Endothelial dysfunction is highly prevalent in HFpEF and is related to reduced exercise capacity and worse outcome [27]. Recently, a new paradigm of HFpEF was suggested, which attributes an important role of comorbidities for myocardial dysfunction and remodelling in HFpEF (Fig. 3; [28]).

## The new paradigm of HFpEF

The new HFpEF paradigm proposes that comorbidities drive structural and functional remodelling in HFpEF through systemic endothelial inflammation [28]. Because of this proinflammatory state, coronary microvascular endothelial cells produce reactive oxygen species, which limits NO bioavailability for adjacent cardiomyocytes. Impaired NO bioavailability results in downregulation of sGC-mediated cGMP-PKG signalling, which augments cardiomyocyte stiffness through hypophosphorylation of titin and increases cardiomyocyte hypertrophy because of impaired PKG-mediated antihypertrophic activity [28]. Furthermore, coronary microvascular endothelial inflammation favours subendothelial migration of leukocytes, which stimulates myofibroblast formation and interstitial collagen deposition. Both increased cardiomyocyte stiffness and interstitial fibrosis induce diastolic LV dysfunction [28].

Microvascular endothelial inflammation is also associated with myocardial capillary rarefaction [29], which was recently demonstrated in HFpEF myocardium [18].

## Myocardial capillary rarefaction

A recent autopsy study demonstrated reduced myocardial capillary density (MCD) in HFpEF patients regardless of the severity of epicardial coronary disease, while the severity of myocardial fibrosis was inversely associated with MCD [18]. Both fibrosis and MCD were similar in those with or without hypertension, suggesting that comorbidities other than hypertension may perpetuate these alterations. Indeed, diabetes mellitus [30] and obesity [30, 31] are known to be associated with MCD. In a recent study performing histological analysis of non-ischaemic myocardium from 57 patients undergoing coronary artery bypass graft surgery, obese patients had lower MCD [31]. Increased body mass index (BMI) was associated with higher pulmonary capillary wedge pressure (PCWP) and lower MCD was associated with both BMI and increased PCWP [31]. Myocardial capillary rarefaction in HFpEF may contribute to decreased maximal myocardial blood flow, impaired oxygen delivery and insufficient metabolic efficiency, which contribute to diastolic LV dysfunction and a higher risk of heart failure in obese individuals [31].

## Heterogeneity in HFpEF

**Fig. 4:**
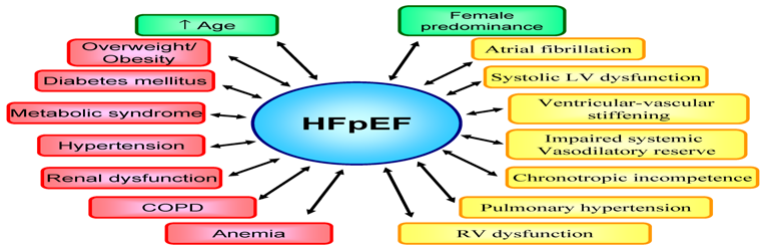
HFpEF represents a heterogeneous syndrome, characterised by multiple cardiovascular and non-cardiovascular comorbidities

HFpEF is difficult to define as illustrated by various diagnostic classifications and inclusion criteria of clinical trials [8]. These factors contributed to heterogeneity of HFpEF patients recruited into trials and registries. Previously, no consensus was present on the optimal LVEF cut-off value and different cut-offs have been used across classifications and trials ranging from LVEF ≥ 40 % to > 50 % [8]. Although diastolic LV dysfunction represents the dominant abnormality in HFpEF, ancillary mechanisms may also contribute, such as systolic LV dysfunction [32, 33], ventricular-vascular stiffening [34], impaired systemic vasodilatory reserve [35], chronotropic incompetence [33, 35], pulmonary hypertension [36] and right ventricular (RV) dysfunction (Fig. 4; [37, 38]).

### Systolic dysfunction

Although ejection fraction is preserved, both chamber and myocardial contractility were subtly but significantly depressed in HFpEF, compared with hypertensive and healthy controls [32]. The extent of myocardial contractile dysfunction in HFpEF related to increased mortality, suggesting that it may be a mediator or marker of more severe disease [32]. Even mild limitations in basal contractility may become more problematic with exercise stress [32, 33]. During exercise, compared with controls, HFpEF patients demonstrated reductions in peak maximal oxygen uptake, chronotropic competence and relative increment in stroke volume and cardiac output [33]. Time to peak diastolic filling increased in HFpEF patients, while it decreased in the control group [33].

### Ventricular-vascular stiffening

Maintaining low ventricular and arterial elastance allows a dynamic range of volume transfer to be achieved during ejection with minimal change in pressure. Ventricular and vascular stiffening increase with ageing, hypertension and diabetes and are abnormally elevated in HFpEF patients [34, 39]. Combined ventricular-arterial stiffening elevates blood pressure lability, amplifying blood pressure changes for any alteration in preload or afterload. Furthermore, combined ventricular-arterial stiffening may also compromise endothelial-dependent vasorelaxation [39].

### Impaired systemic vasodilatory reserve

Patients with HFpEF display attenuated exercise-mediated reductions in mean vascular resistance and arterial elastance, coupled with abnormalities in endothelial function and dynamic ventricular-arterial coupling compared with hypertensive subjects and controls [35]. The extent of impaired flow-mediated vasodilation is related to exercise intolerance [35]. The healthy endothelium has antiproliferative and anti-inflammatory actions and regulates vascular tone by balancing production of vasodilators and vasoconstrictors in response to a variety of stimuli [40]. In a prospective cohort study, endothelial dysfunction was highly prevalent in HFpEF patients and independently correlated with future cardiovascular events [27].

### Chronotropic incompetence

Chronotropic reserve is depressed in HFpEF [33, 35], which could be related to downstream deficits in β-adrenergic stimulation, because the increase in plasma catecholamines with exercise is similar in HFpEF and healthy controls [41]. Autonomic dysfunction may contribute to chronotropic incompetence, as baroreflex sensitivity is reduced and heart rate recovery impaired in HFpEF [41].

### Pulmonary hypertension

In a community-based study, the prevalence of pulmonary hypertension, defined as pulmonary artery systolic pressure (PASP) > 35 mmHg, amounted to 83 % with a median PASP of 48 mmHg in a group of 244 HFpEF patients. In this study, PASP was significantly higher in HFpEF patients than in hypertensives without heart failure, whereas PASP strongly predicted mortality in HFpEF [36]. Chronic elevation of LV filling pressures induces LA remodelling and dysfunction, mixed pulmonary hypertension and ultimately, right ventricular (RV) remodelling and dysfunction [38].

### Right ventricular dysfunction

In an invasive study, right and left heart filling pressures, pulmonary artery pressures and right-sided chamber dimensions were higher in HFpEF compared with controls, while LV size and ejection fraction were similar. RV dysfunction was present in 33 % of HFpEF patients and was caused by both RV contractile impairment and afterload mismatch from pulmonary hypertension [37]. RV dysfunction was also associated with symptom severity and greater comorbidity burden [37]. In a prospective study, approximately one-third of HFpEF patients had evidence of RV dysfunction and both reduced LV compliance and RV dysfunction and remodelling were the strongest pathophysiological predictors of adverse outcomes [38].

## Phenotyping of HFpEF patients

The underlying phenotypic heterogeneity is likely far greater in HFpEF than in HFrEF and may be an important reason for the failure of HFpEF clinical trials [42]. For instance, HFpEF patients with pulmonary hypertension and RV systolic dysfunction responded favourably to the phosphodiesterase type 5 inhibitor sildenafil [43], whereas sildenafil exerted no benefit in HFpEF patients without concomitant RV dysfunction [6]. Recently, phenomapping analysis using statistical learning algorithms demonstrated that HFpEF patients recruited according to uniform diagnostic criteria could be divided into three main distinct subgroups, which differed markedly in clinical characteristics, cardiac structure and function, invasive haemodynamics and outcome, despite similar end-systolic and end-diastolic elastances on LV pressure-volume analysis [42]. Therefore, understanding the phenotypic heterogeneity of HFpEF, which includes the aetiological and pathophysiologic heterogeneity of the syndrome, may allow more targeted and successful HFpEF clinical trials.

## Identifying the severity of myocardial dysfunction and remodelling in HFpEF

**Fig. 5:**
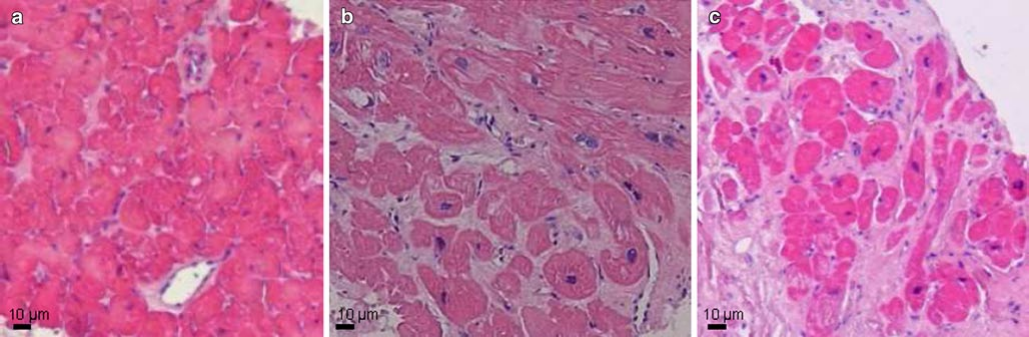
Distinct stages of structural myocardial disease in HFpEF. **a–c**, histological images of LV myocardium from HFpEF patients, demonstrating minor (**a**), moderate (**b**) and severe (**c**) interstitial fibrosis

Recent HFpEF trials demonstrated structural cardiac remodelling in many HFpEF patients including concentric LV remodelling and hypertrophy (59–77 %) and left atrial (LA) dilatation (59–66 %) [5, 7]. Recruitment of LA contractility during stress is impaired in HFpEF and may contribute to the transition from an asymptomatic state to HFpEF, while LA size also predicts clinical outcome [44]. Furthermore, translational studies investigating myocardial tissue from HFpEF patients demonstrated varying degrees of cardiomyoycte hypertrophy, interstitial fibrosis and capillary rarefaction [11, 12, 18], implying distinct and possibly evolutionary stages of myocardial disease progression. Although all large HFpEF trials addressing renin-angiotensin-aldosterone system (RAAS) inhibitors failed to reach statistical significance for the primary outcome, many of them reached borderline significance for a primary endpoint or statistical significance for secondary endpoints, subgroups or post-hoc analyses [3–5, 7, 45, 46]. Because of these findings, the involvement of RAAS in HFpEF appears more subtle than in HFrEF, probably requiring up-front identification of subgroups of HFpEF patients rather than a ‘one fits all strategy’, which has hitherto been applied in HFpEF in analogy to HFrEF. Such a stratification approach with identification of myocardial structural and functional abnormalities in individual HFpEF patients could be relevant for the determination of potential therapeutic responsiveness and selection of appropriate treatment strategies. For instance, the efficacy of RAAS inhibitors to improve adverse myocardial remodelling is likely very different in HFpEF patients with minor, modest or severe stages of myocardial hypertrophy, fibrosis and capillary rarefaction (Fig. 5; [12]). Indeed, RAAS inhibitors might be effective in HFpEF patients presenting at an early stage, whereas therapeutic efficacy may be lost in HFpEF patients presenting at advanced or final stages. AF is likely to emerge as an indicator of advanced disease in HFpEF. A recent subgroup analysis of HFpEF patients recruited in the RELAX trial indeed showed presence of AF to be indicative of longstanding HFpEF [47]. In this study, HFpEF patients with AF were older than those in sinus rhythm, but had similar symptom severity, comorbidities, and renal function. Despite comparable LV size and mass, AF was associated with worse systolic (lower EF, stroke volume, and cardiac index) and diastolic (shorter deceleration time and larger left atria) function compared with sinus rhythm. Patients with AF had higher PASP and increased NT-proBNP, aldosterone, endothelin-1, troponin I, and C-telopeptide for type I collagen levels, suggesting more severe neurohumoral activation, myocyte necrosis, and fibrosis [47]. Multi-biomarker assessment and cardiac imaging modalities could represent promising tools for diagnostic stratification to identify the underlying stage of myocardial disease in the individual HFpEF patient.

## Identifying stage of myocardial disease

### Biomarkers

Currently, only NPs are routinely used for diagnosis and risk stratification in HFpEF patients. Because HFpEF is characterised by inflammation, oxidative stress, endothelial dysfunction, alterations in intramyocardial signalling and matrix remodelling and capillary rarefaction, biomarkers reflecting these processes could aid in diagnostic and pathophysiological stratification in HFpEF patients. Recently, several biomarkers were identified, which appeared to be promising diagnostic and prognostic tools in patients with HFpEF (Table 2; [48]).

**Table 2:** Potential biomarkers for identification of underlying disease processes in HFpEF

Pathophysiological process	Biomarkers
Inflammation	CRP, IL-6, IL-8, IL-10, TNF-α, Pentraxin-3, Galectin-3, MCP-1, GDF-15, Soluble ST2
Extracellular matrix remodelling	MMPs, TIMPs, Collagen propeptides (PICP, PINP, PIIINP, CITP, Galectin-3
Myocyte stress	(NT-pro)BNP, ANP, (nt-proCNP), GDF-15
Myocyte injury/apoptosis	Troponins, Galectin-3
Endothelial dysfunction	E-selectin, P-selectin, VCAM-1, ICAM-1, (NT-proCNP), cGMP
Oxidative stress	Nitrotyrosine
Renal dysfunction	Cystatin-c, microalbuminuria
Miscellaneous	Homocysteine, advanced glycation end products

*CRP* C-reactive protein, *IL-6* interleukin 6, *IL-8* interleukin 8, *IL-10* interleukin 10, *MCP-1* monocyte chemoattractant protein 1, *GDF-15* growth differentiation factor 15, *MMPs* matrix metalloproteinases, *TIMPs* tissue inhibitor of MMPs, *PICP* C-terminal propeptide of procollagen type 1, *PINP* N-terminal propeptide of procollagen type 1, *PIIINP* N-terminal propeptide of procollagen type III, *CITP* C-terminal telopeptide of collagen type I, *ICAM-1* intercellular adhesion molecule 1.

### Imaging

Cardiac magnetic resonance imaging with T1 mapping represents a novel technique, which quantifies the myocardial extracellular volume and that significantly correlates with histological myocardial interstitial fibrosis and LV stiffness [49]. In HFpEF patients, reduced T1 times, indicative of increased interstitial myocardial fibrosis, was associated with worse outcome [50]. Hence, biomarker- and/or imaging-guided quantification of myocardial interstitial fibrosis could aid in selection of HFpEF patients for specific therapeutic strategies.

## Conclusion

HFpEF is a complex and heterogeneous clinical syndrome, which is associated with multiple comorbidities, pathogenic mechanisms and lack of effective treatment. Over the past decade, significant progress has been made in understanding HFpEF pathophysiology and recognising the importance of comorbidities and disease heterogeneity. Importantly, the conceptual framework of HFpEF treatment may need to shift from a ‘one fits all’ strategy to an individualised approach based on phenotypic patient characterisation and diagnostic and pathophysiological stratification of myocardial disease processes. This could ultimately provide an inroad for improved identification of specific treatment targets and enhance development of ‘personalised therapy’ for HFpEF patients.

### Funding

This work was supported by a grant from the European Commission (FP7-Health-2010; MEDIA-261409).
